# Integrated Network Pharmacology and Molecular Dynamics Reveal Luteolin from *Persea americana* as a Multi-Cancer SRC/GSK3β Inhibitor

**DOI:** 10.3390/ijms27146534

**Published:** 2026-07-22

**Authors:** Akey Krishna Swaroop, Bharat Kumar Reddy Sanapalli, Jubie Selvaraj, Dilep Kumar Sigalapalli, Ramya Tokala, Vidyasrilekha Sanapalli

**Affiliations:** 1 Department of Pharmaceutical Chemistry, Shri Vile Parle Kelavani Mandal’s College of Pharmacy, Shirpur 425421, MH, India; krishna.akey@svkm.ac.in; 2 Department of Pharmacology, School of Pharmacy & Technology Management, SVKM’s Narsee Monjee Institute of Management Studies (NMIMS) Deemed-to-be-University, Jadcherla 509301, TS, India; bharathsanapalli@yahoo.in; 3 Department of Pharmaceutical Chemistry, JSS College of Pharmacy, JSS Academy of Higher Education & Research, The Nilgiris, Ooty 643001, TN, India; jubie@jssuni.edu.in; 4 Department of Biochemistry, University of Washington, Seattle, WA 98195, USA; dilep@uw.edu; 5 Department of Radiology, Athinoula A. Martinos Center for Biomedical Imaging, Massachusetts General Hospital and Harvard Medical School, Charlestown, MA 02129, USA; 6 Department of Pharmaceutical Chemistry, School of Pharmacy & Technology Management, SVKM’s Narsee Monjee Institute of Management Studies (NMIMS) Deemed-to-be-University, Jadcherla 509301, TS, India

**Keywords:** *Persea americana*, multi-cancer, luteolin, network pharmacology, molecular dynamics

## Abstract

Cancer progression is driven by dysregulated kinase signaling and apoptotic evasion across multiple malignancies. Although targeted kinase inhibitors have improved outcomes, resistance and toxicity remain major challenges. Natural phytochemicals offer promising multi-target therapeutic potential. *Persea americana* contains diverse bioactive compounds; however, its role in multi-cancer kinase targeting remains underexplored. This study aimed to identify and validate anti-cancer kinase targets of *Persea americana* phytoconstituents across five cancers: lung, breast, cervical, colorectal, and prostate, using integrated network pharmacology and molecular simulation approaches. Cancer-associated genes were retrieved from the Open Targets Platform and prioritized through Gene Ontology analysis. Overlapping targets with 208 predicted human targets of *Persea americana* were identified. Protein–protein interaction networks revealed hub genes, followed by TCGA-based validation. Twenty-five phytoconstituents were docked against SRC and GSK3β, and top complexes underwent 100 ns molecular dynamics simulations. Enrichment highlighted kinase activity and apoptosis. SRC emerged as a pan-cancer hub, while GSK3β was prominent in breast cancer. Luteolin showed strongest binding to SRC (−11.9 kcal/mol), outperforming the co-crystal inhibitor, while valencene showed affinity toward GSK3β (−8.8 kcal/mol). Simulations confirmed stable interactions. Luteolin exhibits strong multi-target kinase inhibition, particularly against SRC, supporting its potential as a pan-cancer therapeutic candidate.

## 1. Introduction

Cancer remains one of the leading causes of morbidity and mortality worldwide, with a rapidly expanding burden driven by population aging, lifestyle transitions, and persistent health disparities [1,2]. According to the latest GLOBOCAN 2022 estimates, nearly 20 million new cancer cases and 9.7 million cancer-related deaths were recorded globally, with lung, breast, colorectal, and prostate cancer collectively accounting for close to 40% of total cancer incidence worldwide [3]. In the United States, the American Cancer Society projects approximately 2.04 million new cancer cases and 618,120 cancer-related deaths in 2025, with a marked shift toward higher incidence among women and middle-aged adults, alongside sustained mortality from smoking and lifestyle associated malignancies [4]. Similarly, data from the National Cancer Registry Programme of India estimate a rise in cancer cases from 1.39 million in 2020 to nearly 1.57 million by 2025, highlighting a substantial and accelerating cancer burden in a developing-country context [5].

The United States and India were chosen as comparative epidemiological contexts because both represent contrasting high-income and rapidly transitioning healthcare systems– and both countries have complementary perspectives on cancer burden due to lifestyle factors versus disparity- and infection-driven cancer burden. This study is complemented by global age-standardised data that provide a context to the individualities of the five malignancies studied. Breast cancer is the most common cancer in women globally, and is the most common cancer in both American and Indian female populations, with the high incidence and the increasing therapeutic resistance driving a need for new multi-target therapeutic agents [3]. Despite a great deal of progress in the treatment of targeted therapy, lung cancer remains the most lethal cancer and accounts for almost 19% of cancer mortality worldwide due to its aggressive nature and persistently poor five-year survival despite advances in targeted therapy. The incidence of colorectal cancer (CRC) has been steadily increasing among younger and middle-aged populations, and this is owing to Westernised dietary and sedentary lifestyle practices, which are also a feature of urban Indians and the US population. Prostate cancer is one of the five most prevalent cancers worldwide, and in the United States, the incidence of prostate cancer is higher in older men, and an increase in detection has been reported in India with the increased use of PSA testing for prostate cancer screening. Despite its largely preventable nature, despite vaccination and screening, cervical cancer is also a disproportionately heavy burden in India, where it makes up about 11–12% of female cancers, and is also clinically relevant in the US, although its absolute incidence has significantly decreased over time due to screening and HPV vaccination, with the burden still high amongst underserved populations [4,6]. Together, these five cancers account for the majority of cancer burden in the world in terms of incidence and mortality, share several dysregulated kinase-driven signalling pathways, and disproportionately affect both wealthy and less affluent countries, so for this study, they were chosen as a representative, multi-cancer panel.

Natural products have historically played a pivotal role in oncology drug discovery, providing structurally diverse bioactive scaffolds capable of multi-target modulation [7]. *Persea americana* (avocado), a member of the Lauraceae family native to Central America and now cultivated worldwide, has gained attention beyond its nutritional value due to its rich phytochemical composition [8,9]. The fruit contains flavonoids, terpenoids, polyphenols, carotenoids, and tocopherols that exhibit antioxidant, anti-inflammatory, antimicrobial, and anticancer activities [10,11]. Bioactive compounds such as luteolin and various sesquiterpenoids have demonstrated promising pharmacological effects, including modulation of oxidative stress and inhibition of proliferative signaling pathways [12]. Its long-standing traditional medicinal use and emerging evidence in inflammatory and chronic diseases further support its therapeutic relevance [13]. Of these, luteolin has been well-studied as an anticancer flavonoid in various cancers, and has been shown to affect apoptosis, angiogenesis, metastasis and cell cycle regulation [14]. Independent evidence has emerged that luteolin is a multi-target anticancer agent, in non-small-cell lung cancer, targeting Akt/MDM2/p53 pathway [15], and in colorectal/colon carcinoma, targeting the AKT1/SRC/MDK3 pathway [16]. Luteolin has also recently been demonstrated to inhibit two pathways linked to GSK3β in estrogen receptor-positive breast cancer stem cells [17]. There has been, however, no study that has comprehensively assessed luteolin, or phytoconstituents in Persea americana generally, against a wide variety of epidemiologically prominent cancers within an all-encompassing network pharmacology and molecular dynamics approach.

Given the escalating burden of major cancers and the need for safer multi-target therapeutic strategies, the present study was designed to systematically evaluate the anticancer potential of *Persea americana* phytoconstituents against five epidemiologically dominant malignancies breast, lung, colorectal, prostate, and cervical cancers. By integrating network pharmacology, transcriptomic validation, molecular docking, and molecular dynamics simulations, this work aims to identify key kinase targets shared across cancers and to elucidate the mechanistic potential of avocado-derived compounds in multi-cancer therapeutic intervention.

In the common set of hub genes found across the five cancers (Section 2.4), SRC and GSK3β were chosen for detailed structural and dynamic study due to their strong position in the protein–protein interaction networks, and to their strong association with tumour-associated transcriptional dysregulation supported by expression analyses from the TCGA (Section 2.5). Both kinases are known regulators of oncogenic signalling: SRC is known to contribute to proliferation, invasion, and chemoresistance in a variety of epithelial malignancies [18], while GSK3β acts as a context-dependent tumor suppressor and oncogenic molecule in the regulation of Wnt/β-catenin and PI3K/Akt signaling [19]. Given their convergent dysregulation in breast, lung, colorectal, prostate and cervical cancers, along with their known druggability, they were prioritized as multi-cancer therapeutic targets in this study.

## 2. Results

### 2.1. Cancer-Associated Gene Dataset

Target retrieval from the Open Targets Platform yielded a substantial number of genes associated with each selected cancer type. A total of 15,702 genes were identified for lung cancer, 17,229 genes for breast cancer, 8452 genes for cervical cancer, 15,057 genes for colorectal cancer, and 11,229 genes for prostate cancer. Among the five cancer types, The reported differences in the number of cancer-associated genes reflect the inherent molecular heterogeneity and pathological characteristics of the selected tumor types. Breast cancer exhibited the highest number of associated genes, which is consistent with its well-recognized molecular heterogeneity comprising multiple intrinsic subtypes and diverse signaling pathways. In contrast, cervical cancer showed the lowest number of associated genes, reflecting its comparatively more defined disease biology. Thus, the variation in the number of retrieved targets likely represents differences in the molecular complexity of each cancer type rather than methodological bias. These biological aspects have been considered in interpreting the retrieved gene datasets were subsequently subjected to functional prioritization and gene ontology enrichment analysis using the ToppGene Suite to identify biologically relevant and druggable targets.

### 2.2. Functional Enrichment and Target Prioritization

GO enrichment analysis revealed highly significant overrepresentation of protein kinase activity, regulation of apoptotic process, and cell surface components across all five cancer types. The enrichment remained statistically robust after Benjamini–Hochberg correction, with extremely low FDR values ranging from 10^−36^ to 10^−164^, confirming strong functional clustering rather than random gene overlap. Across cancer types, Lung cancer retained 725 kinase-related genes (FDR: 2.74 × 10^−38^), 1604 apoptosis-regulating genes (FDR: 5.21 × 10^−110^), and 1002 cell-surface genes (FDR: 3.92 × 10^−86^). Breast cancer retained 767 kinase genes, 1662 apoptotic regulators, and 1021 cell-surface genes. Cervical cancer retained 533 kinase genes, 1241 apoptotic regulators, and 716 cell-surface genes. Colorectal cancer retained 740 kinase genes, 1586 apoptotic regulators, and 982 cell-surface genes. Prostate cancer retained 636 kinase genes, 1447 apoptotic regulators, and 844 cell-surface genes. The consistent enrichment of these categories across all five malignancies indicates a convergent oncogenic architecture centered on dysregulated kinase signaling, apoptotic escape, and membrane-associated therapeutic targets. The conserved enrichment of these GO categories across all five cancer types suggests that, despite differences in tissue origin and pathological characteristics, they share fundamental molecular mechanisms involved in tumor development and progression. Protein kinase activity represents a central regulatory component of oncogenic signaling pathways, while dysregulation of apoptotic processes enables sustained tumor cell survival and therapeutic resistance. Similarly, the enrichment of cell surface-associated proteins highlights their importance in tumor–microenvironment interactions and their accessibility as potential therapeutic targets. Therefore, the consistent enrichment of these biologically relevant and pharmacologically actionable categories provided a strong rationale for prioritizing genes for subsequent overlap analysis, protein–protein interaction network construction, and molecular docking studies. Accordingly, these prioritized gene subsets were subsequently used for intersection analysis with Persea americana constituent-associated targets.

### 2.3. Identification of Common Genes Between Persea americana and Cancer Targets

**Figure 1:**
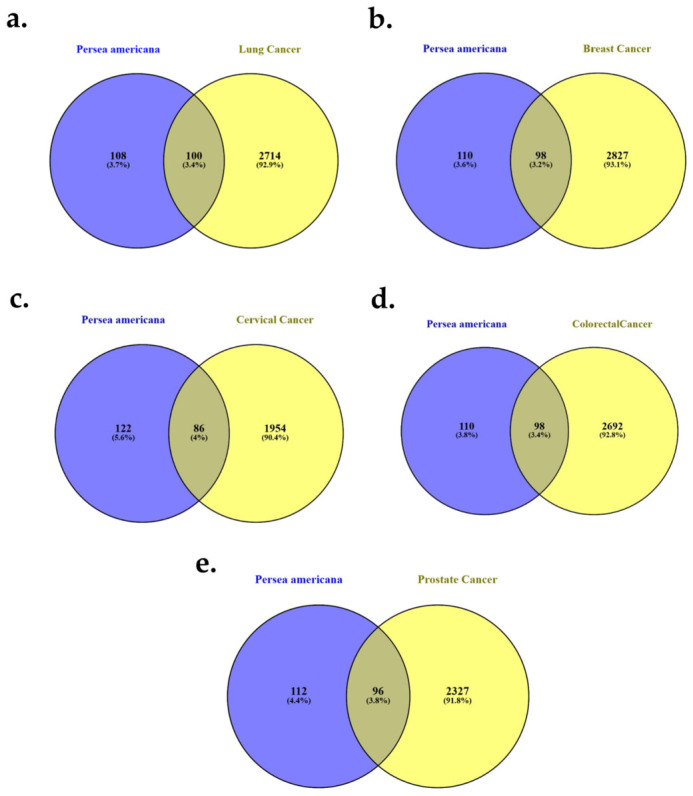
The Venn diagram-based integrative analysis identified a number of intersecting genes among *Persea americana* targets and, (**a**). lung, (**b**). breast, (**c**). cervical, (**d**). colorectal, and (**e**). prostate cancer gene sets, suggesting some overlapping molecular pathways that could be targeted for multi-cancer drug development.

Following functional prioritization, the finalized non-redundant gene sets for each cancer type were intersected with the 208 predicted human targets of *Persea americana* to identify biologically relevant shared genes. Venn diagram analysis was performed using the Venny 2.1 web-based platform. Independent pairwise comparisons were conducted for lung, breast, cervical, colorectal, and prostate cancers (Figure 1a–e). The intersection analysis revealed 100 common genes for lung cancer (2814 final genes), 98 for breast cancer (2925 genes), 86 for cervical cancer (2040 genes), 98 for colorectal cancer (2790 genes), and 96 for prostate cancer (2423 genes). Despite the large size of cancer-associated gene pools, a consistent subset of overlapping genes was observed across all five malignancies. Importantly, approximately 3–4% of the prioritized cancer-associated genes overlapped with *Persea americana* targets in each comparison, suggesting selective enrichment rather than random or non-specific gene overlap. The reproducible presence of shared genes across multiple cancer types indicates a conserved molecular interaction landscape and supports the hypothesis that *Persea americana* may exert multi-cancer therapeutic effects through common regulatory mechanisms. These overlapping genes were therefore considered biologically significant and were selected for subsequent protein–protein interaction network construction and hub gene identification. The comprehensive workflow of cancer target collection, functional prioritization, duplicate removal, and identification of common genes between *Persea americana* and each cancer type is summarized in Table 1.

### 2.4. Protein–Protein Interaction Network Analysis and Hub Gene Identification

The PPI network analysis demonstrated complex interaction patterns among the common drug–disease targets across all five cancer types. The number of nodes and edges varied slightly between cancers, reflecting differences in network density and interaction complexity. For lung cancer, the PPI network constructed from 100 common genes comprised 100 nodes and 723 edges, indicating a highly interconnected network. CytoNCA analysis identified EGFR (degree = 54, betweenness = 1137.800881, closeness = 0.405738) as the most central hub gene, followed by SRC, ESR1, HSP90AB1, and GSK3β. In breast cancer, the network contained 98 nodes and 717 edges. Similar to lung cancer, EGFR showed the highest degree (54) along with strong betweenness (1112.444) and closeness (0.409283) values. SRC, ESR1, HSP90AB1, and GSK3β were also consistently ranked among the top five hub genes. For cervical cancer, 86 common genes generated a network with 86 nodes and 652 edges. EGFR again emerged as the top hub gene (degree = 51, closeness = 0.416667), followed by ESR1, SRC, HSP90AB1, and MMP9. The presence of MMP9 among the top hubs suggests a cancer-specific variation in cervical cancer network topology. In colorectal cancer, the PPI network consisted of 98 nodes and 721 edges. EGFR remained the dominant hub gene (degree = 54), followed by SRC, ESR1, HSP90AB1, and GSK3β, indicating strong conservation of key oncogenic signaling mediators across gastrointestinal malignancies. For prostate cancer, 96 common genes formed a network with 96 nodes and 700 edges. EGFR (degree = 53) was again the most connected node, followed by SRC, ESR1, HSP90AB1, and GSK3β. Across all five cancers, EGFR, SRC, ESR1, and HSP90AB1 consistently appeared as central hub genes, while GSK3β was dominant in four cancers and MMP9 was specific to cervical cancer. The identified hub genes are well-established regulators of multiple oncogenic processes and further support the biological relevance of the present network pharmacology findings. EGFR and SRC are key mediators of cell proliferation, survival, migration, and metastasis through activation of major signaling pathways [18,20], whereas HSP90AB1 functions as a molecular chaperone that stabilizes numerous oncogenic client proteins involved in tumor progression [21]. ESR1 contributes to hormone-dependent tumor development through estrogen-mediated signaling [22], while GSK3β regulates several cancer-associated pathways, including Wnt/β-catenin signaling, apoptosis, and cell proliferation [19]. Interestingly, MMP9 emerged as a unique hub gene in cervical cancer, consistent with its established role in extracellular matrix degradation, tumor invasion, and angiogenesis. This centrality in cervical cancer is mechanistically associated with high-risk HPV oncoproteins E6 and E7, which are known to regulate the transcription of MMP9 in HPV-driven lesions in a way that promotes basement membrane invasion in cervical lesions, but not in the other 4 cancers examined [23].

**Figure 2:**
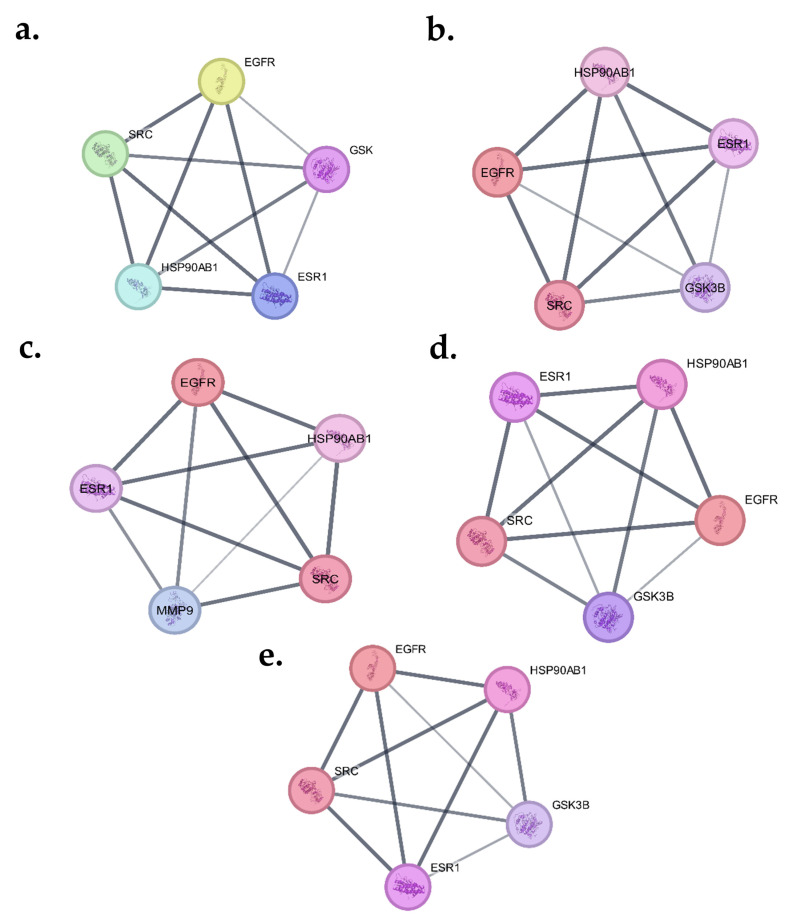
The figure shows the hub genes networks integrated from the merged *Persea americana* targets with gene sets for, (**a**). lung, (**b**). breast, (**c**). cervical, (**d**). colorectal, and (**e**). prostate cancers. Genes are the nodes and edges are protein–protein or functional interactions.

These observations provide biological support for the identified hub genes and further justify their prioritization for subsequent gene expression validation and molecular docking analyses. Figure 2a–e illustrates the top hub gene networks for lung, breast, cervical, colorectal, and prostate cancers, respectively, and Table 2 summarizes the corresponding topological parameters.

### 2.5. Gene Expression Validation and Final Target Prioritization

To refine the hub genes identified from PPI network analysis, gene expression validation was performed using TCGA datasets and visualized through BoxplotR analysis. For each cancer type, the five top-ranked hub genes were comparatively analyzed to identify the most consistently dysregulated and biologically dominant candidate suitable for downstream docking studies. The box plots illustrate the distribution of normalized gene expression values across samples, and the statistical parameters (upper whisker, Q3, median, Q1, and lower whisker) provide quantitative insight into expression variability and central tendency.

#### 2.5.1. Lung Cancer

In lung cancer, SRC (Upper whisker = 2.42; Q3 = 0.54; Median = −0.16; Q1 = −0.73; Lower whisker = −1.95) and GSK3β (Upper whisker = 2.37; Q3 = 0.52; Median = −0.15) demonstrated the widest expression spread among the five genes. The higher Q3 values and extended upper whiskers indicate stronger overexpression tendencies in a subset of samples compared to ESR1 and EGFR, which showed relatively narrow distributions. Although HSP90AB1 also showed moderate variability, SRC displayed the highest upper whisker and one of the highest Q3 values, suggesting greater transcriptional activity in tumor samples. Therefore, SRC was prioritized as the dominant target in lung cancer.

#### 2.5.2. Breast Cancer

In breast cancer, GSK3β (Upper whisker = 2.25; Q3 = 0.51; Median = −0.15; Lower whisker = −2.15) exhibited the broadest expression range and one of the highest third-quartile values among the five genes. ESR1 showed a higher upper whisker (2.12), but its median expression was comparatively lower (−0.32), indicating less consistent elevation across samples. EGFR demonstrated minimal variability (Upper whisker = 0.30), suggesting limited dysregulation. Based on distribution width and Q3 values, GSK3β demonstrated stronger expression dominance, supporting its selection as the key target in breast cancer.

#### 2.5.3. Cervical Cancer

In cervical cancer, SRC (Upper whisker = 2.50; Q3 = 0.59; Median = −0.17; Lower whisker = −2.01) exhibited the highest upper whisker and Q3 value among all five genes. HSP90AB1 also showed moderate spread (Upper whisker = 1.99), but its Q3 was lower compared to SRC. MMP9 and EGFR displayed comparatively narrow ranges, indicating weaker dysregulation patterns. The pronounced expression spread and higher third-quartile value of SRC indicate its stronger involvement in cervical tumor biology. Thus, SRC was prioritized for cervical cancer.

#### 2.5.4. Colorectal Cancer

In colorectal cancer, SRC again showed the highest upper whisker (2.44) and Q3 value (0.56), indicating significant upregulation in a subset of samples. HSP90AB1 demonstrated moderate expression spread (Upper whisker = 1.83; Q3 = 0.46), whereas ESR1 showed minimal variability (Upper whisker = 0.17). EGFR and GSK3β displayed intermediate expression levels. The consistently higher distribution range and quartile values of SRC suggest its central regulatory role in colorectal cancer. Accordingly, SRC was selected as the primary candidate target.

#### 2.5.5. Prostate Cancer

In prostate cancer, SRC (Upper whisker = 2.15; Q3 = 0.49; Median = −0.04) demonstrated the highest expression dispersion and upper-quartile distribution among the analyzed hub genes. HSP90AB1 also exhibited relatively elevated expression (Q3 = 0.36), whereas EGFR, GSK3β, and ESR1 showed comparatively lower expression distributions. The broader variability and higher upper-range expression observed for SRC indicate greater heterogeneity in its expression across prostate cancer samples. Based on these expression characteristics, SRC was prioritized as the most dysregulated and biologically significant target in prostate cancer.

**Figure 3:**
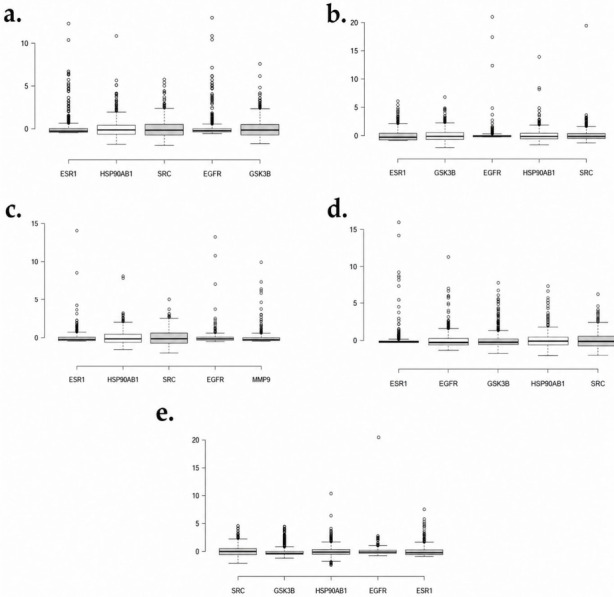
The figure depicts the top 5 highly expressed hub genes, analysis of overlapping between *Persea americana* and gene sets associated with (**a**). lung, (**b**). breast, (**c**). cervical, (**d**). colorectal, and (**e**). prostate cancers.

Across five cancers, SRC consistently exhibited the highest or among the highest upper whisker and Q3 values in lung, cervical, colorectal, and prostate cancers, indicating broad expression variability and strong tumor-associated dysregulation. In breast cancer, GSK3β demonstrated comparatively stronger distribution dominance and was therefore selected as the primary candidate for that cancer type. The consistent recurrence of SRC as a highly expressed hub gene across multiple malignancies highlights its potential as a pan-cancer therapeutic target. Combined with its central position in the PPI network and strong expression variability in TCGA datasets, SRC was identified as the principal molecular target linking *Persea americana* phytoconstituents and multi-cancer pathogenesis, while GSK3β emerged as a cancer-specific alternative target in breast cancer. These prioritized targets were subsequently subjected to molecular docking and molecular dynamics simulations to evaluate the binding potential of selected *Persea americana* bioactive compounds. The comparative gene expression distributions of the top five hub genes across lung, breast, cervical, colorectal, and prostate cancers are illustrated in Figure 3a–e, and the corresponding descriptive statistical parameters obtained from BoxplotR analysis are summarized in Table 3.

### 2.6. Active Site Identification

The active binding pockets of SRC (PDB ID: 3F3V) and GSK3β (PDB ID: 7SXJ) were identified using PLIP based on interactions of their respective co-crystallized ligands. In SRC, key hydrophobic interactions were observed with *Leu273A*, *Glu310A*, *Leu322A*, *Tyr340A*, *Tyr382A*, *Leu393A*, *Val402A*, and *Asp404A*, along with hydrogen bonds involving *Glu310A*, *Met341A*, and *Asp404A*, and π-stacking with *Phe405A*, confirming the ATP-binding catalytic pocket. In GSK3β, the binding site comprised hydrophobic contacts with *Ile62A*, *Val70A*, *Ala83A*, *Tyr134A*, *Thr138A*, and *Leu188A*, while critical hydrogen bonding was observed with *Val135A*. These experimentally validated residues were used to define the docking grid for subsequent molecular docking analysis.

### 2.7. Molecular Docking Results

Molecular docking analysis was performed for 25 selected Persea americana phytoconstituents against GSK3β (PDB ID: 7SXJ) and SRC (PDB ID: 3F3V) using the AutoDock Vina algorithm implemented in PyRx. Binding affinities were calculated in kcal/mol, and lower (more negative) values indicate stronger binding stability.

For GSK3β (7SXJ), the co-crystallized ligand exhibited a binding affinity of −7.7 kcal/mol. Among the phytoconstituents, IMPHY011896 (Valencene, a sesquiterpenoid) demonstrated the strongest binding affinity of −8.8 kcal/mol, surpassing the reference inhibitor. IMPHY004660 (Luteolin, a flavone) also showed a strong binding score of −8.0 kcal/mol. These values indicate favorable binding stability within the GSK3β catalytic pocket compared to the native ligand.

For SRC (3F3V), the co-crystallized inhibitor showed a binding affinity of −9.0 kcal/mol. Notably, IMPHY004660 (Luteolin) demonstrated the highest binding affinity of −11.9 kcal/mol, significantly stronger than the standard inhibitor. IMPHY011896 (Valencene) also exhibited strong binding at −9.8 kcal/mol, exceeding the co-crystal reference. Several other compounds, including IMPHY013080 (−8.4 kcal/mol), IMPHY009718 (−8.1 kcal/mol), and IMPHY004281 (−8.1 kcal/mol), showed moderate binding stability; however, none surpassed the performance of Luteolin.

Overall, Luteolin (IMPHY004660) exhibited the strongest interaction with SRC, while Valencene (IMPHY011896) showed superior binding toward GSK3β. Importantly, both compounds demonstrated binding energies comparable to or better than their respective co-crystallized inhibitors, suggesting stable interaction within the ATP-binding domains. This SRC-binding result is in agreement with another docking and molecular dynamic study that analyzed an independent screening of luteolin against colon and colorectal carcinoma using network pharmacology methods, which showed that SRC was one of the key targets for luteolin in these types of cancer [16]. Luteolin on the other hand, showed excellent binding inside the catalytic pocket of GSK3β and this is consistent with previous biochemical studies which revealed a direct interaction between luteolin and the catalytic site of GSK3β, with an IC_50_ of 1.5 µm and low predicted interaction energy in the same binding pocket as GSK3β [24]. The consistent high affinity of Luteolin toward SRC, combined with the recurrent identification of SRC as a hub gene across multiple cancers, highlights SRC–Luteolin interaction as a promising therapeutic axis. This has been further corroborated by reports of luteolin’s multi-targeted anticancer activity in other network pharmacology studies such as non-small-cell lung cancer by targeting Akt/MDM2/p53 axis [15] and breast cancer stem cells by targeting GSK3β-associated pathways [17] making it a promising multi-cancer kinase targeting phytoconstituent. Therefore, Valencene and Luteolin were selected for further molecular dynamics simulation studies to evaluate complex stability and interaction dynamics over time. The detailed binding affinity values of all 25 screened phytoconstituents against GSK3β and SRC, along with their respective co-crystallized reference inhibitors, are presented in Table 4.

### 2.8. Molecular Dynamics Simulation Analysis

**Figure 4:**
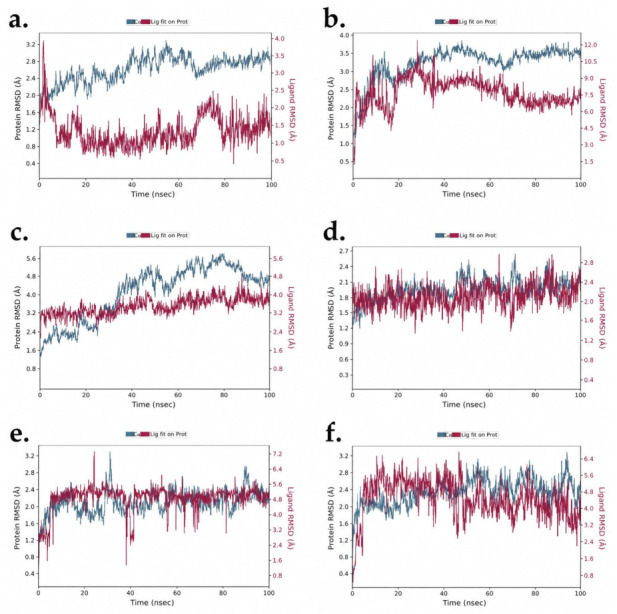
The figure depicts the RMSD profiles obtained from MD simulations performed using Desmond for 100 ns, (**a**). SRC–Luteolin, (**b**). SRC–Valencene, (**c**). SRC–Co-crystal, (**d**). GSK3β–Luteolin, (**e**). GSK3β–Valencene, (**f**). GSK3β-Co-crystal.

To further validate the stability and dynamic behavior of the docked complexes, 100 ns molecular dynamics (MD) simulations were performed for six systems: SRC (PDB ID: 3F3V) complexed with Luteolin, Valencene, and the co-crystal inhibitor (Figure 4a–c), and GSK3β (PDB ID: 7SXJ) complexed with Luteolin, Valencene, and its co-crystal ligand (Figure 4d–f). All simulations were conducted under NPT conditions at 300 K. The structural stability of each complex was evaluated using root mean square deviation (RMSD) analysis of the protein backbone (Cα atoms) and ligand fitted to the protein.

#### 2.8.1. Root Mean Square Deviation (RMSD) Analysis for SRC (3f3v) Complexes and GSK3β (7SXJ) Complexes

For the SRC–Luteolin complex (Figure 4a), the protein backbone RMSD increased during the initial equilibration phase (first 10–15 ns) and subsequently stabilized within 2.2–3.0 Å for the remainder of the simulation. The ligand RMSD remained consistently below 2.1 Å after equilibration, indicating strong positional stability within the active site. These results demonstrate a well-maintained binding conformation throughout the 100 ns trajectory. In contrast, the SRC–Valencene complex (Figure 4b) exhibited moderate protein backbone stability (1.2–3.8 Å); however, the ligand RMSD showed persistent high deviations, reaching values up to ~10 Å and failing to achieve a stable plateau. This suggests significant ligand displacement and weaker binding stability. The SRC–co-crystal complex (Figure 4c) displayed larger protein backbone deviations (1.5–5.8 Å) compared to the Luteolin complex. The ligand RMSD fluctuated between 3.0–4.3 Å, indicating moderate binding stability but greater flexibility relative to Luteolin. Collectively, stability ranking for SRC systems was:

Luteolin > Co−crystal > Valencene

Notably, Luteolin demonstrated superior dynamic stability compared to the native co-crystallized inhibitor.

The GSK3β–Luteolin complex (Figure 4d) exhibited excellent structural stability, with protein backbone RMSD maintained within 1.3–2.5 Å throughout the simulation. The ligand RMSD remained within 1.4–2.8 Å, indicating tight and stable binding within the active site. For the GSK3β–Valencene complex (Figure 4e), the protein backbone remained stable (1.3–3.2 Å); however, the ligand RMSD stabilized around 4.5–5.5 Å after equilibration, suggesting moderate flexibility within the binding pocket. The GSK3β –co-crystal complex (Figure 4f) showed protein RMSD values between 1.5–2.9 Å and ligand RMSD fluctuations primarily within 3.0–5.0 Å, indicating moderate binding stability. For GSK3β systems, the stability ranking was:

Luteolin > Valencene ≈ Co−crystal

Overall, RMSD analysis across all six systems revealed that Luteolin maintained the lowest ligand deviations and the most consistent protein backbone stability for both SRC and GSK3β targets (Table 5). These findings strongly support Luteolin as the most dynamically stable multi-target compound among the tested phytoconstituents.

#### 2.8.2. Root Mean Square Fluctuation (RMSF) Analysis for SRC Complexes & GSK3β Complexes

**Figure 5:**
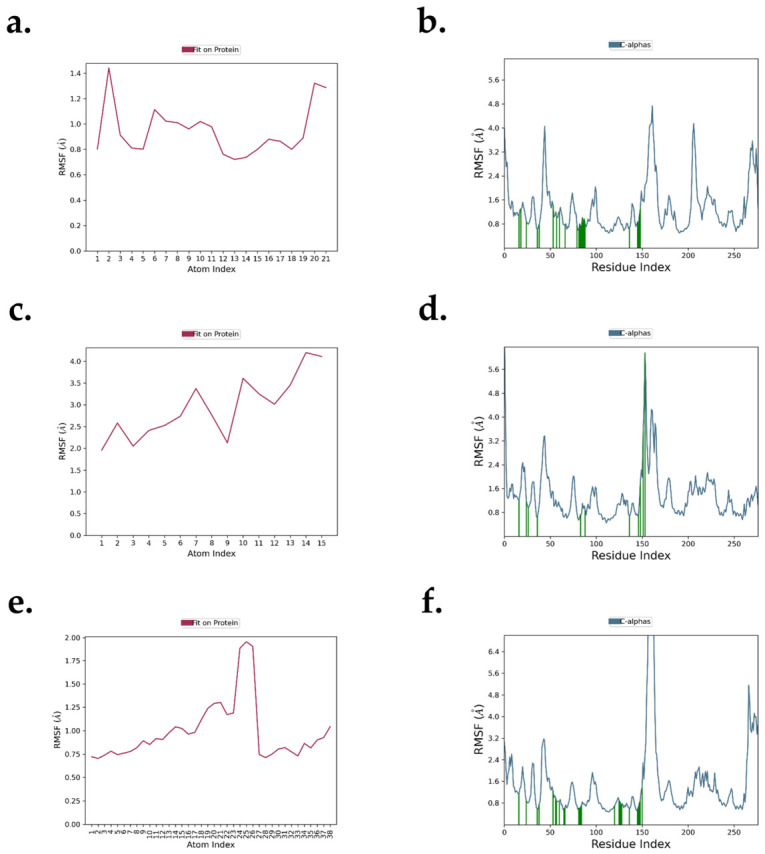
The figure depicts the RMSF profiles of ligand and protein obtained from MD simulations performed using Desmond for 100 ns, SRC–Luteolin (**a**,**b**), SRC–Valencene (**c**,**d**), SRC–Co-crystal (**e**,**f**).

The RMSF profiles of SRC complexes (Figure 5a–f) were evaluated to understand residue-level flexibility and ligand stability throughout the MD simulation. In the SRC–Luteolin complex (Figure 5a,b), the Cα atoms predominantly fluctuated within 0.5–1.8 Å, indicating a stable backbone conformation. Minor peaks observed in surface loop regions did not affect the structural integrity of the catalytic site. The ligand RMSF values remained within ~1.0–2.7 Å, suggesting moderate but controlled internal flexibility within the binding pocket. For the SRC–Valencene complex (Figure 5c,d), the protein backbone showed slightly increased fluctuations compared to luteolin, with localized peaks approaching ~3.0 Å in flexible loop regions. The ligand exhibited comparatively higher atomic deviations (~2.0–4.2 Å), indicating greater conformational mobility and weaker pocket confinement. In the SRC–co-crystal system (Figure 5e,f), the backbone RMSF values were largely maintained below 1.6 Å, except for a pronounced peak around residue ~30–35 corresponding to a surface-exposed loop. The active site residues displayed minimal fluctuation (<1.5 Å), confirming preservation of catalytic architecture. Ligand RMSF values ranged between ~1.0–2.2 Å, reflecting stable binding behavior comparable to the reference system. Overall, among SRC complexes, luteolin demonstrated improved binding stability compared to valencene, with RMSF behavior close to the co-crystallized ligand.

**Figure 6:**
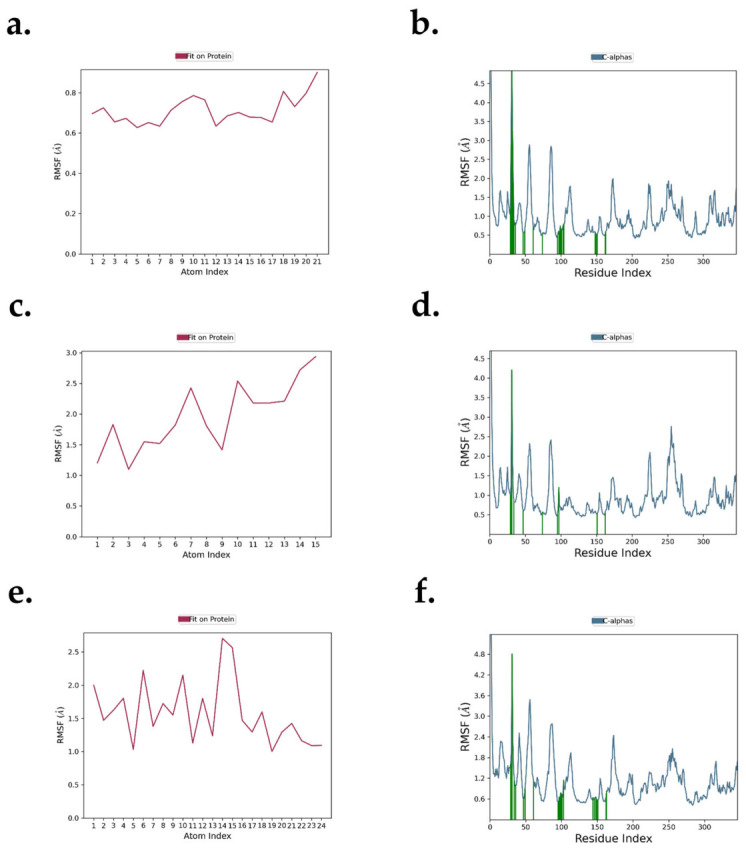
The figure depicts the RMSF profiles of ligand and protein obtained from MD simulations performed using Desmond for 100 ns of, GSK3β –Luteolin (**a**,**b**), GSK3β–Valencene (**c**,**d**), GSK3β–Co-crystal (**e**,**f**).

The RMSF patterns of GSK3β complexes (Figure 6a–f) revealed consistent backbone stability with localized flexibility in loop regions. In the GSK3β–Luteolin complex (Figure 6a,b), most Cα residues fluctuated within 0.5–1.5 Å, except for a loop region near residue ~30–35 exhibiting higher deviation (~4.5–4.8 Å). Importantly, ATP-binding site residues maintained low RMSF values (<1.5 Å), indicating stable ligand accommodation. The ligand RMSF ranged approximately between 0.7–0.9 Å, suggesting strong confinement within the catalytic pocket. For the GSK3β–Valencene complex (Figure 6c,d), the backbone fluctuations were slightly elevated in certain regions (~2.0–2.8 Å), while the ligand displayed higher internal motion (~1.1–2.9 Å), reflecting reduced stabilization relative to luteolin. In the GSK3β–co-crystal complex (Figure 6e–f), backbone RMSF values were predominantly below 1.6 Å, with a notable surface loop fluctuation (~4.8 Å). The catalytic core remained structurally preserved, and ligand deviations (~1.0–2.7 Å) were moderate and controlled. Collectively, the GSK3β systems indicate that luteolin exhibits the lowest ligand mobility and strongest active-site stabilization, followed by the co-crystal reference, while valencene shows comparatively higher flexibility.

Comparative RMSF evaluation between SRC and GSK3β indicates that the GSK3β–Luteolin complex exhibited the lowest overall residue and ligand fluctuations, reflecting superior structural stability among all systems. Across both kinases, backbone deviations remained minimal with only localized loop flexibility and no significant disturbance at the active sites. Notably, luteolin consistently demonstrated lower ligand mobility than valencene and stability comparable to or better than the co-crystal references. These findings highlight the strong conformational stability of luteolin within oncogenic kinase targets, supporting its potential relevance in cancer-targeted therapeutic development.

#### 2.8.3. Protein–Ligand Contact Analysis During MD Simulation SRC and GSK3β Complexes

**Figure 7:**
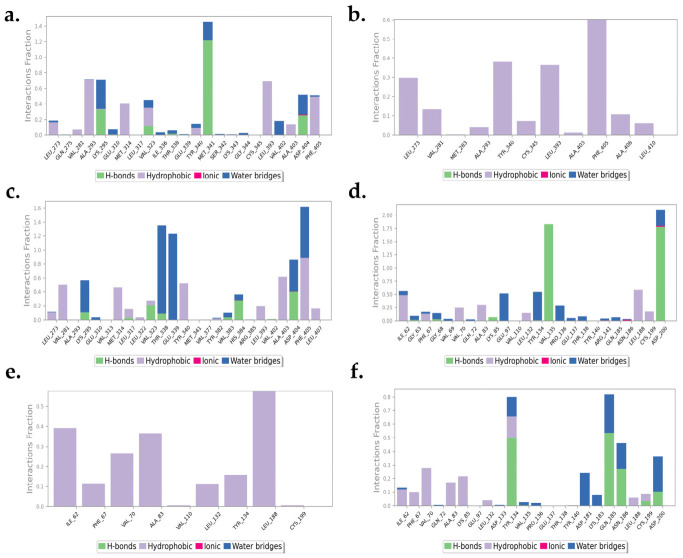
The figure depicts the protein-ligand contact profiles obtained from MD simulations performed using Desmond for 100 ns, (**a**). SRC–Luteolin, (**b**). SRC–Valencene, (**c**). SRC–Co-crystal, (**d**). GSK3β–Luteolin, (**e**). GSK3β–Valencene, (**f**). GSK3β-Co-crystal.

The protein–ligand contact analysis of SRC complexes confirms stable occupation of the ATP-binding catalytic pocket throughout the MD simulation. In the SRC–Luteolin complex (Figure 7a), strong and persistent interactions were observed with key catalytic and hinge-region residues including Met341, Lys295, Ala293, Asp404, and Phe405. Notably, Met341 and Asp404 exhibited high interaction fractions, supporting stable hydrogen bonding within the hinge region. Aromatic stabilization with Phe405 and hydrophobic contacts involving Leu273, Val281, Leu322, Tyr340, Leu393, and Val402 further confirm tight ligand confinement within the ATP-binding cleft. In contrast, the SRC–Valencene complex (Figure 7b) demonstrated predominantly hydrophobic interactions, mainly with Phe405, Tyr340, Leu393, and Leu273, but with reduced interaction fractions and minimal hinge-region hydrogen bonding. Limited engagement with Asp404 and Met341 suggests weaker catalytic stabilization compared to luteolin. The SRC–Co-crystal complex (Figure 7c) showed conserved hinge interactions involving Thr338 and Glu339, along with strong catalytic engagement of Asp404 and Phe405. The interaction pattern closely resembles that of luteolin, validating active-site integrity and simulation reliability. Overall, within SRC systems, luteolin exhibited interaction persistence and hinge stabilization comparable to the co-crystal reference and superior to valencene.

The GSK3β–Luteolin complex (Figure 7d) displayed strong hinge-region interactions with Val135 and Tyr134, alongside significant engagement of Asp200 in the activation loop. Additional contacts with Ile62, Ala83, Lys85, and Pro136 confirm sustained ATP-pocket occupancy. The high cumulative interaction fractions indicate robust dynamic stabilization within the catalytic cleft. The GSK3β–Valencene complex (Figure 7e) primarily formed hydrophobic interactions with Leu188, Ile62, Ala83, and Val70, but showed limited hinge-region engagement and minimal interaction with critical catalytic residues such as Asp200. This reduced interaction persistence aligns with the higher ligand flexibility observed in RMSD and RMSF analyses. In the GSK3β–Co-crystal complex (Figure 7f), stable hinge interaction with Tyr134 and consistent engagement of Gln185, Asn186, and Asp200 were maintained, confirming preserved catalytic architecture. The interaction pattern served as a structural benchmark, with luteolin demonstrating comparable or enhanced stabilization. Across both SRC and GSK3β kinase systems, luteolin consistently maintained strong hinge-region hydrogen bonding and sustained catalytic residue engagement, closely resembling or exceeding co-crystal interaction patterns. Valencene, while retaining hydrophobic contacts, lacked stable hinge anchoring and showed reduced interaction persistence. Collectively, the protein–ligand contact analysis confirms that luteolin achieves superior active-site confinement and dynamic stability in both oncogenic kinase targets, reinforcing its potential relevance in cancer-directed therapeutic strategies.

## 3. Discussion

Cancer is a multifactorial disease driven by complex molecular dysregulation involving aberrant kinase signaling, apoptotic escape, and altered cell-surface communication. In the present study, a comprehensive network pharmacology and molecular simulation approach was employed to explore the multi-cancer therapeutic potential of *Persea americana* (avocado) phytoconstituents. Initially, large-scale cancer-associated gene datasets were retrieved for lung, breast, cervical, colorectal, and prostate cancers. Functional enrichment analysis consistently highlighted significant overrepresentation of protein kinase activity, regulation of apoptosis, and cell-surface components across all malignancies, confirming a convergent oncogenic architecture. Intersection analysis between prioritized cancer genes and 208 predicted human targets of *Persea americana* identified a reproducible subset of shared genes in each cancer type, supporting selective and biologically meaningful overlap rather than random association. Protein–protein interaction network analysis further refined these candidates and consistently identified EGFR, SRC, ESR1, HSP90AB1, and GSK3β as central hub genes. Subsequent TCGA-based gene expression validation revealed that SRC exhibited dominant dysregulation in lung, cervical, colorectal, and prostate cancers, while GSK3β showed stronger expression prominence in breast cancer. These findings prioritized SRC as a pan-cancer target and GSK3β as a critical complementary kinase target. Active-site mapping of SRC (3F3V) and GSK3β (7SXJ) confirmed canonical ATP-binding catalytic residues, which guided molecular docking of 25 avocado-derived phytoconstituents. Luteolin demonstrated the strongest binding affinity toward SRC (−11.9 kcal/mol), surpassing the co-crystal inhibitor, while valencene showed superior binding toward GSK3β (−8.8 kcal/mol). Molecular dynamics simulations over 100 ns further validated complex stability, where luteolin consistently exhibited lower RMSD values, faster equilibration, reduced RMSF, and strong hinge-region confinement compared to valencene and even co-crystal references. Protein–ligand contact analysis confirmed persistent engagement of key catalytic residues in both kinases, particularly for luteolin, which maintained stable hydrogen bonding and hydrophobic interactions within the ATP-binding pockets. Overall, this integrative workflow from large-scale cancer target identification and network prioritization to docking and dynamic simulation demonstrates that avocado-derived luteolin exhibits strong multi-target stability against SRC and GSK3β, two central oncogenic kinases. These findings highlight *Persea americana* phytoconstituents as promising candidates for further experimental validation and development as multi-cancer therapeutic agents.

## 4. Materials and Methods

### 4.1. Cancer-Associated Target Identification

Cancer-associated genes were retrieved from the Open Targets Platform (https://platform.opentargets.org/ accessed on 7 February 2026), a comprehensive integrative resource that links genes to diseases using genetic, transcriptomic, clinical, and literature-based evidence. Five major cancers were selected based on global incidence and mortality statistics: lung cancer, breast cancer, cervical cancer, colorectal cancer, and prostate cancer. For each cancer type, the disease name was queried individually within the Open Targets Platform [25]. All genes associated with the respective cancer were downloaded without applying additional evidence score filtering to ensure comprehensive target coverage at this stage. Gene lists were exported in standardized gene symbol format for downstream analysis. The total number of genes retrieved for each cancer type was recorded for subsequent enrichment and network analyses [26,27].

### 4.2. Functional Gene Prioritization and Gene Ontology Enrichment

To prioritize biologically relevant and therapeutically actionable targets, the retrieved cancer-associated gene sets were subjected to functional enrichment analysis using the ToppGene Suite [28]. Gene Ontology (GO) enrichment was performed across three major ontological domains: Molecular Function (MF), Biological Process (BP), and Cellular Component (CC). Among significantly enriched GO terms, three functionally and pharmacologically relevant categories were selected for downstream prioritization [29,30].

#### 4.2.1. Molecular Function

Protein kinase activity (GO:0004672), Protein kinases represent one of the most druggable protein families in oncology. Their well-defined ATP-binding catalytic domains provide structurally conserved pockets amenable to small-molecule inhibition. Kinase dysregulation drives oncogenic signaling, proliferation, and metastasis across multiple cancer types. Given that kinase inhibitors constitute a substantial proportion of approved targeted anticancer therapies, enrichment of this category supports translational feasibility [31,32].

#### 4.2.2. Biological Process

Regulation of apoptotic process (GO:0042981), Evasion of apoptosis is a fundamental hallmark of cancer. Genes involved in apoptotic regulation directly influence tumor survival, therapeutic resistance, and disease progression. Targeting apoptotic pathways restores programmed cell death and represents a core mechanism of anticancer therapy [33,34].

#### 4.2.3. Cellular Component

Cell surface (GO:0009986), Cell surface proteins are readily accessible to therapeutic intervention, including small molecules, monoclonal antibodies, and antibody-drug conjugates. Selection of this category enhances clinical translatability by prioritizing targets that overcome intracellular delivery limitations [35,36].

Significantly enriched genes within these GO categories were retained based on Benjamini–Hochberg false discovery rate (FDR) correction, ensuring robust statistical confidence and minimizing type I error. Only terms with highly significant adjusted FDR values were considered for further analysis [37].

### 4.3. Identification of Bioactive Constituents and Target Genes of Persea americana

Phytochemical constituents of *Persea americana* were retrieved from the IMPPAT database as previously reported by Akey et al. [38]. A total of 253 phytocompounds (98 fruit-derived and 155 leaf-derived) were collected and structurally prepared for screening. ADMET-based filtering was performed using Data Warrior to identify compounds with favorable drug-likeness and safety profiles based on molecular weight (<500 Da), logP (−2 to 5), logS (>−5), hydrogen bond acceptors (≤10), hydrogen bond donors (≤5), rotatable bonds (≤10), polar surface area (<140 Å^2^), and absence of predicted mutagenic, tumorigenic, irritant, or reproductive toxicity risks. Following this screening, 64 phytoconstituents met the selection criteria. Target prediction for these compounds was conducted using BindingDB with a similarity threshold ≥ 0.5. Twenty-five compounds yielded predicted human targets, which were standardized using the UniProt database (*Homo sapiens*). After removal of duplicates, a final set of 208 unique gene targets associated with *Persea americana* was obtained. While these phytochemical and target identification steps were previously established in the context of rheumatoid arthritis, the current study newly integrates these validated *Persea americana* targets with multi-cancer gene datasets to investigate potential anticancer mechanisms.

### 4.4. Identification of Common Genes Between Persea americana and Selected Cancers

The final non-redundant gene sets obtained for each cancer type after functional prioritization were intersected with the previously identified 208 human targets of *Persea americana*. The overlap analysis was performed using the Venny 2.1 web-based tool [39,40]. Separate pairwise comparisons were conducted for lung, breast, cervical, colorectal, and prostate cancers to determine shared genes between avocado-associated targets and cancer-specific prioritized genes.

### 4.5. Protein–Protein Interaction (PPI) Network Construction and Hub Gene Identification

To investigate the interaction landscape of the intersected drug–disease targets, protein–protein interaction (PPI) networks were constructed for each cancer type using the STRING database. The lists of common genes obtained from the Venn analysis (intersection between *Persea americana* targets and functionally prioritized cancer genes) were imported into STRING with the organism restricted to *Homo sapiens*. A minimum interaction confidence score of 0.4 (medium confidence) was applied for network construction, and disconnected nodes were excluded to retain only interacting proteins for subsequent network analysis. The interaction data were then exported and visualized using Cytoscape software (version 3.10.3) [41,42]. Topological analysis of the networks was performed using the CytoNCA plugin in Cytoscape to identify hub genes based on three centrality parameters: degree centrality, betweenness centrality, and closeness centrality [43]. Degree centrality reflects the number of direct interactions a node has and is commonly used to identify hub proteins that play critical roles in network stability and signal propagation. Betweenness centrality measures the extent to which a node lies on the shortest paths between other nodes, indicating its regulatory control over information flow. Closeness centrality represents how close a node is to all other nodes in the network, reflecting its potential to rapidly influence other proteins. For each cancer-specific PPI network, genes were ranked according to Degree centrality, and the top five genes with the highest Degree values were selected as hub genes. The corresponding Betweenness and Closeness centrality values were used to further support their topological importance [44]. These hub genes were subsequently subjected to gene expression validation using TCGA datasets and box plot analysis to identify the most relevant therapeutic target for downstream molecular docking studies.

### 4.6. Gene Expression Validation and Target Prioritization Using TCGA and BoxplotR

To further prioritize the top five hub genes identified from protein–protein interaction network analysis, gene expression validation was performed using The Cancer Genome Atlas (TCGA) database through the Genomic Data Commons (GDC) Data Portal. The “Access TCGA Data” option was utilized, followed by selection of the “Analysis Center” interface for web-based analysis and visualization. For each cancer type-lung, breast, cervical, colorectal, and prostate, the corresponding TCGA dataset was selected individually [45,46]. Within the analysis module, the gene expression clustering option was accessed. Although predefined gene clusters were available within the platform, these were modified to include only the five top-ranked hub genes identified from the respective PPI networks. These five genes were uploaded for each cancer type independently. Following submission, gene expression data were downloaded in tab-separated values (TSV) format. The expression values obtained from TCGA were then subjected to further visualization and statistical interpretation using the BoxplotR web server. The TSV files were converted to CSV format where required and uploaded through the “Data Upload” option in BoxplotR [47]. The platform generated graphical representations of expression distribution in the form of box plots along with corresponding descriptive statistical tables, including upper whisker, lower whisker, quartiles, and median values. The comparative expression distribution patterns of the five hub genes were analyzed for each cancer type to identify the most consistently dysregulated and statistically relevant target. The gene demonstrating stronger expression alteration and distribution significance was selected as the final prioritized target for subsequent molecular docking and molecular dynamics simulation studies [44].

### 4.7. Molecular Docking Studies

Molecular docking analysis was performed to evaluate the binding affinity of selected *Persea americana* phytoconstituents against the prioritized cancer targets SRC and GSK3β. Based on gene expression validation, SRC was selected as the principal multi-cancer target and GSK3β as a cancer-specific target. A total of 25 phytoconstituents previously identified through ADMET filtering and target prediction were selected for docking analysis [38].

#### 4.7.1. Protein Preparation

The three-dimensional crystal structure of SRC kinase domain was retrieved from the Protein Data Bank (PDB ID: 3F3V), resolved by X-ray diffraction at a resolution of 2.60 Å. This structure represents the kinase domain of c-SRC in complex with a Type II inhibitor (RL45). The co-crystallized ligand, 1-{4-[(6-aminoquinazolin-4-yl)amino]phenyl}-3-[3-tert-butyl-1-(3-methylphenyl)-1H-pyrazol-5-yl]urea, was used as the reference standard for docking validation. For GSK3β, the crystal structure of the BIO-2895 (BRD0705)-bound GSK3β–axin complex was retrieved (X-ray diffraction, resolution 1.85 Å). The co-crystallized ligand, (4S)-4-ethyl-7,7-dimethyl-4-phenyl-2,6,8,9-tetrahydropyrazolo [3,4-b]quinolin-5-one, was used as the standard reference inhibitor. Protein preparation was performed using Swiss-PdbViewer v4.1. Co-crystallized ligands, water molecules, and heteroatoms were removed, and the protein structures were cleaned. Energy minimization was conducted to optimize the protein geometry, and the prepared structures were saved in PDB format for subsequent docking studies [48,49].

#### 4.7.2. Ligand Preparation

The selected 25 phytoconstituents of *Persea americana* were obtained from the IMPPAT database. The ligand structures were imported into PyRx software and converted into PDBQT format. Energy minimization was performed within PyRx to ensure optimized ligand conformations prior to docking [50].

#### 4.7.3. Active Site Identification and Receptor Grid Generation

The active binding pockets of SRC and GSK3β were identified using the Protein–Ligand Interaction Profiler (PLIP) by analyzing the interaction residues of the respective co-crystallized ligands. Docking was restricted to these experimentally validated active sites to ensure biologically relevant binding predictions. The SRC binding pocket was predominantly hydrophobic, comprising Leu273, Leu322, Tyr340, Tyr382, Leu393, Val402, and Phe405, while Glu310, Met341, and Asp404 contributed key polar interactions involved in ligand stabilization. Similarly, the GSK3β ATP-binding pocket exhibited a predominantly hydrophobic environment formed by Ile62, Val70, Ala83, Tyr134, and Leu188, whereas Val135 and Thr138 served as important polar residues involved in hydrogen-bond formation. The estimated cavity volume and solvent-accessible surface area of the SRC binding pocket were 34,800 Å^3^ and 201.215 Å^2^, respectively, whereas the GSK3β binding pocket exhibited a cavity volume of 45,991.8 Å^3^ and a surface area of 319.601 Å^2^, both calculated using a 1.4 Å probe radius. Grid box parameters were defined to encompass the catalytic pocket region surrounding the co-crystallized ligand binding site [51].

#### 4.7.4. Docking Procedure

Molecular docking simulations were performed using PyRx 0.8 incorporating the AutoDock Vina algorithm. The prepared proteins were designated as macromolecules and the ligands as flexible docking molecules. Docking was executed at the predefined active site grid coordinates. For SRC (PDB ID: 3F3V), the docking grid center was defined at X = −5.3453, Y = 32.1493, and Z = −7.1123 with grid dimensions of 27.7987 × 29.7813 × 19.6933 Å. For GSK3β (PDB ID: 7SXJ), the docking grid center was defined at X = 14.5178, Y = −8.7638, and Z = 32.3713 with grid dimensions of 19.8999 × 22.4837 × 21.3098 Å. The exhaustiveness parameter was set to 8 for all docking simulations. Both the 25 phytoconstituents and the corresponding co-crystallized standard inhibitors were docked under identical parameters for comparative analysis. Binding affinities were calculated in kcal/mol, and docking poses were ranked based on minimum binding energy. Compounds exhibiting stronger binding affinity than or comparable to the co-crystal standard were selected for further molecular dynamics simulation studies to evaluate complex stability and interaction behavior over time [52,53].

### 4.8. Molecular Dynamics Simulation

To further validate the stability of the docked complexes, molecular dynamics (MD) simulations were performed for six protein–ligand systems: SRC–Valencene, SRC–Luteolin, SRC–co-crystal inhibitor (PDB ID: 3F3V), and GSK3β–Valencene, GSK3β–Luteolin, GSK3β–co-crystal inhibitor (PDB ID: 7SXJ). SRC was selected as the prioritized hub target for lung, breast, colorectal, and prostate cancers, while GSK3β was selected as the key target for cervical cancer based on gene expression and docking outcomes. The docked complexes obtained from PyRx were used as initial structures for MD simulations. Simulations were carried out using the Desmond module of the Schrödinger Suite. The OPLS3e force field was applied for accurate molecular interaction modeling [54]. The systems were solvated using the TIP4P explicit water model within an orthorhombic simulation box, maintaining a 10 Å buffer distance between the protein complex and box boundaries. Energy minimization was performed using the OPLS3e force field with 200 steps of steepest descent followed by 1000 steps of the conjugate gradient method until convergence at a gradient threshold of 25 kcal/mol [55]. Electrostatic interactions were calculated using the smooth particle mesh Ewald (SPME) method with a short-range cutoff of 9.0 Å and long-range tolerance of 1 × 10^−9^. All simulations were conducted under the NPT ensemble at 300 K temperature and 1 bar pressure [44]. A Nose–Hoover thermostat and Martyna–Tobias–Klein barostat were used to maintain system stability. Each system was simulated for 100 nanoseconds with a time step of 2 femtoseconds. The reversible reference system propagator algorithm (RESPA) was employed for integrating bonded and non-bonded interactions. These simulations were performed to evaluate the structural stability, dynamic behavior, and binding persistence of the selected phytocompounds within the active sites of SRC and GSK3β. Comparative analysis of RMSD, RMSF, hydrogen bonding was used to determine the most stable complex and identify the most promising therapeutic candidate for multi-cancer targeting.

## 5. Conclusions

In this study, we employed an integrated systems pharmacology and molecular simulation framework to elucidate the multi-cancer therapeutic potential of *Persea americana* phytoconstituents. By combining large-scale cancer target mining, functional gene prioritization, intersection analysis, network topology evaluation, transcriptomic validation, molecular docking, and long-timescale molecular dynamics simulations, we systematically narrowed thousands of cancer-associated genes to two mechanistically central kinase targets SRC and GSK3β. Functional enrichment across five major malignancies consistently revealed convergence on dysregulated protein kinase activity, apoptotic control pathways, and membrane-associated signaling, underscoring a shared oncogenic architecture. Intersection analysis demonstrated selective and reproducible overlap between cancer-associated genes and predicted *Persea americana* targets, supporting biologically meaningful multi-cancer relevance. Network centrality analysis identified SRC and GSK3β as dominant regulatory hubs, and TCGA-based gene expression validation further confirmed SRC as a pan-cancer dysregulated target, with GSK3β exhibiting cancer-specific prominence in breast cancer. Structure-based docking demonstrated that luteolin and valencene exhibit binding affinities comparable to or exceeding co-crystallized inhibitors within the ATP-binding catalytic pockets of SRC and GSK3β. Importantly, molecular dynamics simulations over 100 ns provided mechanistic validation, revealing that luteolin maintains superior structural stability, sustained hinge-region hydrogen bonding, minimal backbone perturbation, and persistent catalytic-site engagement across both kinase systems. Protein–ligand contact analyses further confirmed conserved interaction with key catalytic residues, reinforcing the reliability of the docking predictions. Collectively, these findings suggest that luteolin from *Persea americana* is a computationally predicted, dynamically stable multi-target kinase inhibitor with potential pan-cancer applicability. The convergence of network centrality, transcriptomic dysregulation, high binding affinity, and long-term conformational stability provides strong computational evidence supporting its potential therapeutic relevance. However, these findings require further experimental validation through in vitro and in vivo studies. This work advances the understanding of avocado-derived phytochemicals in oncology and demonstrates a robust integrative computational framework for rational multi-cancer drug discovery. The identified phytoconstituents, particularly luteolin, represent potential lead compounds for kinase-targeted cancer therapeutics based on computational predictions. Nevertheless, these findings should be considered hypothesis-generating and require comprehensive in vitro and in vivo experimental validation before their therapeutic potential can be confirmed.

## Figures and Tables

**Table 1:** Functional Prioritization and Identification of Common Genes Between *Persea americana* and Selected Cancers.

Cancer Type	Total Genes (Open Targets)	GO: Protein Kinase Activity (FDR; Genes)	GO: Regulation of Apoptotic Process (FDR; Genes)	GO: Cell Surface (FDR; Genes)	Total Genes After GO Merge	Final Non-Redundant Genes	*Persea americana* Targets	Common Genes (Venn)
Lung	15,702	2.74 × 10^−38^; 725	5.21 × 10^−110^; 1604	3.92 × 10^−86^; 1002	3331	2814	208	100
Breast	17,229	1.73 × 10^−36^; 767	1.39 × 10^−87^; 1662	6.80 × 10^−63^; 1021	3450	2925	208	98
Cervical	8452	1.26 × 10^−46^; 533	2.44 × 10^−158^; 1241	4.77 × 10^−75^; 716	2490	2040	208	86
Colorectal	15,057	1.94 × 10^−52^; 740	4.44 × 10^−112^; 1586	1.67 × 10^−81^; 982	3308	2790	208	98
Prostate	11,229	5.40 × 10^−52^; 636	4.37 × 10^−164^; 1447	1.61 × 10^−79^; 844	2927	2423	208	96

**Table 2:** Topological parameters of the top five hub genes identified from protein–protein interaction (PPI) network analysis of common drug–disease targets across five cancers.

Cancer	Nodes	Edges	Top 5 Genes	Degree	Closeness	Betweenness
Lung cancer	100	723	EGFR	54	0.405738	1137.800881
			SRC	52	0.402439	879.7040955
			ESR1	49	0.39759	763.6688848
			HSP90AB1	45	0.391304	849.0363492
			GSK3β	42	0.385214	485.2389727
Breast cancer	98	717	EGFR	54	0.409283	1112.444
			SRC	52	0.405858	866.3456
			ESR1	49	0.400826	750.964
			HSP90AB1	45	0.392713	736.2881
			GSK3β	42	0.388	475.7386
Cervical cancer	86	652	EGFR	51	0.416667	758.5731
			ESR1	48	0.410628	607.4496
			SRC	46	0.406699	429.412
			HSP90AB1	44	0.400943	499.9863
			MMP9	40	0.395349	474.4428
Colorectal cancer	98	721	EGFR	54	0.411017	1122.243
			SRC	52	0.405858	851.1486
			ESR1	49	0.400826	739.1261
			HSP90AB1	45	0.392713	728.6256
			GSK3β	42	0.388	464.9648
Prostate cancer	96	700	EGFR	53	0.411255	1076.599
			SRC	50	0.404255	746.6794
			ESR1	49	0.402542	737.8449
			HSP90AB1	45	0.394191	726.7002
			GSK3β	41	0.387755	453.8826

**Table 3:** Descriptive statistical parameters of hub gene expression across five cancers (BoxplotR analysis).

Cancer Type	Gene	Upper Whisker	Q3	Median	Q1
Lung	ESR1	0.66	0.02	−0.29	−0.40
	HSP90AB1	1.97	0.41	−0.14	−0.63
	SRC	2.42	0.54	−0.16	−0.73
	EGFR	0.57	0.01	−0.22	−0.37
	GSK3β	2.37	0.52	−0.15	−0.72
Breast	ESR1	2.12	0.38	−0.32	−0.79
	GSK3β	2.25	0.51	−0.15	−0.69
	EGFR	0.30	−0.01	−0.14	−0.21
	HSP90AB1	1.86	0.40	−0.16	−0.60
	SRC	1.63	0.33	−0.18	−0.53
Cervical	ESR1	0.72	0.04	−0.28	−0.41
	HSP90AB1	1.99	0.43	−0.20	−0.63
	SRC	2.50	0.59	−0.17	−0.72
	EGFR	0.56	0.05	−0.17	−0.35
	MMP9	0.55	−0.01	−0.29	−0.41
Colorectal	ESR1	0.17	−0.07	−0.18	−0.23
	EGFR	1.60	0.29	−0.26	−0.59
	GSK3β	1.33	0.21	−0.25	−0.55
	HSP90AB1	1.83	0.46	−0.09	−0.58
	SRC	2.44	0.56	−0.11	−0.71
Prostate	ESR1	1.65	0.29	−0.29	−0.64
	EGFR	1.02	0.19	−0.14	−0.37
	GSK3β	0.79	−0.01	−0.36	−0.54
	HSP90AB1	1.67	0.36	−0.15	−0.54
	SRC	2.15	0.49	−0.04	−0.64

**Table 4:** Molecular Docking Binding Affinities of *Persea americana* Phytoconstituents Against 7SXJ (GSK3β) and 3F3V (SRC).

Ligand	Compound Name	Binding Affinity with GSK3β (PDB ID: 7SXJ) (kcal/mol)	Binding Affinity with SRC (PDB ID: 3F3V) (kcal/mol)
IMPHY011896	Valencene	−8.8	−9.8
IMPHY004660	Luteolin	−8	−11.9
136980453 (Cocrystal)	(4~{S})-4-ethyl-7,7-dimethyl-4-phenyl-2,6,8,9-tetrahydropyrazolo [3,4-b]quinolin-5-one	−7.7	42601396 (1-{4-[(6-aminoquinazolin-4-yl)amino]phenyl}-3-[3-tert-butyl-1-(3-methylphenyl)-1H-pyrazol-5-yl]urea)(Cocrystal)—9
IMPHY010640	Eudesmol	−6.9	−7.8
IMPHY008936	alpha-Guaiene	−6.8	−7.5
IMPHY011659	alpha-Muurolene	−6.8	−7.7
IMPHY011542	beta-Eudesmol	−6.8	−7.4
IMPHY009718	Bulnesol	−6.8	−8.1
IMPHY011589	7-epi-alpha-Eudesmol	−6.7	−7.3
IMPHY013080	alpha-Calacorene	−6.7	−8.4
IMPHY011792	gamma-Muurolene	−6.7	−7.9
IMPHY004281	Guaiol	−6.7	−8.1
IMPHY014885	1-Isopropyl-4,7-dimethyl-1,3,4,5,6,8a-hexahydro-4a(2H)-naphthalenol	−6.4	−7.5
IMPHY012586	(-)-alpha-Cadinol	−6.3	−7
IMPHY015128	T-Muurolol	−6.2	−8
IMPHY017739	4′-Methoxybutyrophenone	−5.7	−6.1
IMPHY011058	3-Cyclohexen-1-ol, 4-methyl-1-(1-methylethyl)-, acetate	−5.6	−6.3
IMPHY006149	4′-Methoxyacetophenone	−5.4	−5.6
IMPHY011396	4-Carvomenthenol	−5.3	−5.8
IMPHY011590	d-Borneol	−5.3	−6
IMPHY011515	4-Methoxybenzoic acid	−5.2	−5.4
IMPHY012036	Camphor	−5.2	−6
IMPHY015249	2-Dodecanol	−4.7	−5.1
IMPHY006992	1-Methylhexyl acetate	−4.5	−5
IMPHY009626	2-Nonanol	−4.5	−4.9
IMPHY007357	Nicotinic acid	−4.5	−4.9

**Table 5:** RMSD Analysis Summary of 100 ns Molecular Dynamics Simulations.

Complex	Protein RMSD Range (Å)	Ligand RMSD Range (Å)	Equilibration Time (ns)	Stability Assessment
SRC–Luteolin	1.8–3.2	0.6–2.1	10–15	Highly Stable
SRC–Valencene	1.2–3.8	1.5–10.0	Not clearly achieved	Unstable
SRC–Co-crystal	1.5–5.8	3.0–4.3	~20	Moderately Stable
GSK3β–Luteolin	1.3–2.5	1.4–2.8	10–15	Highly Stable
GSK3β–Valencene	1.3–3.2	1.0–5.8	~15	Moderately Stable
GSK3β–Co-crystal	1.5–2.9	0.5–5.8	~15	Moderately Stable

## Data Availability

No new data were created or analyzed in this study. Data sharing is not applicable to this article.

## Abbreviations

The following abbreviations are used in this manuscript:

ATP	Adenosine Triphosphate
ADMET	Absorption, Distribution, Metabolism, Excretion, and Toxicity
BP	Biological Process
CC	Cellular Component
Cα	Alpha Carbon
CytoNCA	Cytoscape Network Centrality Analysis Plugin
EGFR	Epidermal Growth Factor Receptor
ESR1	Estrogen Receptor 1
FDR	False Discovery Rate
GDC	Genomic Data Commons
GEPIA	Gene Expression Profiling Interactive Analysis
GO	Gene Ontology
GSK3Β	Glycogen Synthase Kinase 3 Beta
HSP90AB1	Heat Shock Protein 90 Alpha Family Class B Member 1
IMPPAT	Indian Medicinal Plants, Phytochemistry and Therapeutics Database
MD	Molecular Dynamics
MF	Molecular Function
MMP9	Matrix Metalloproteinase 9
NPT	Constant Number of Particles, Pressure, and Temperature Ensemble
OPLS3e	Optimized Potentials for Liquid Simulations 3e Force Field
PDB	Protein Data Bank
PLIP	Protein Ligand Interaction Profiler
PPI	Protein–Protein Interaction
RESPA	Reversible Reference System Propagator Algorithm
RMSD	Root Mean Square Deviation
RMSF	Root Mean Square Fluctuation
SPME	Smooth Particle Mesh Ewald
SRC	Proto Oncogene Tyrosine Protein Kinase Src
STRING	Search Tool for the Retrieval of Interacting Genes/Proteins
TCGA	The Cancer Genome Atlas
TIP4P	Transferable Intermolecular Potential with 4 Points Water Model
TSV	Tab-Separated Values
UniProt	Universal Protein Resource
